# Effect of fluid overload on survival in patients with sepsis-induced acute kidney injury receiving continuous renal replacement therapy

**DOI:** 10.1038/s41598-023-29926-w

**Published:** 2023-02-16

**Authors:** Il Young Kim, Suji Kim, Byung Min Ye, Min Jeong Kim, Seo Rin Kim, Dong Won Lee, Hyo Jin Kim, Harin Rhee, Sang Heon Song, Eun Young Seong, Soo Bong Lee

**Affiliations:** 1 Department of Internal Medicine, Pusan National University School of Medicine, Yangsan, South Korea; 2 Research Institute for Convergence of Biomedical Science and Technology, Pusan National University Yangsan Hospital, Yangsan, South Korea; 3 Medical Research Institute, Pusan National University Hospital, Busan, South Korea

**Keywords:** Acute kidney injury, Continuous renal replacement therapy

## Abstract

The association between fluid overload and survival has not been well elucidated in critically ill patients with sepsis-induced acute kidney injury (SIAKI) receiving continuous renal replacement therapy (CRRT). We investigated the optimal cutoff value of fluid overload for predicting mortality and whether minimizing fluid overload through CRRT is associated with a survival benefit in these patients. We examined 543 patients with SIAKI who received CRRT in our intensive care unit. The degree of cumulative fluid overload in relation to body weight was expressed as the percentage fluid overload (%FO). %FO was further subdivided into %FO from AKI diagnosis to CRRT initiation (%FOpreCRRT) and total fluid overload (%FOtotal). The best cutoff value of fluid overload for predicting the 28-day mortality was %FOpreCRRT > 4.6% and %FOtotal > 9.6%. Multivariable analysis demonstrated that patients with %FOpreCRRT > 4.6% and %FOtotal > 9.6% were 1.9 times and 3.37 times more likely to die than those with %FOpreCRRT ≤ 4.6% and %FOtotal ≤ 9.6%. The 28-day mortality was the highest in patients with %FOpreCRRT > 4.6% and %FOtotal > 9.6% (84.7%), followed by those with %FOpreCRRT ≤ 4.6% and %FOtotal > 9.6% (65.0%), %FOpreCRRT > 4.6% and %FOtotal ≤ 9.6% (43.6%), and %FOpreCRRT ≤ 4.6% and %FOtotal ≤ 9.6% (22%). This study demonstrated that fluid overload was independently associated with the 28-day mortality in critically ill patients with SIAKI. Future prospective studies are needed to determine whether minimizing fluid overload using CRRT improves the survival of these patients.

Acute kidney injury (AKI) is a common and serious complication that occurs in over 50% of critically ill patients^1^. Mortality among critically ill patients with AKI has been reported to be more than 50% and as high as 80% in patients requiring renal replacement therapy (RRT)^2–4^. Sepsis is the leading cause of AKI in patients admitted to the intensive care unit (ICU), accounting for approximately 50% of all cases of AKI^3,5^.

In critically ill patients, hemodynamic instability requires aggressive fluid resuscitation, which leads to fluid overload. In patients with sepsis, sepsis itself contributes to the development of fluid overload. Inflammatory cascading reactions including a variety of mediators that occurs in sepsis increase microvascular permeability and capillary leakage which, in turn, result in interstitial fluid accumulation, loss of protein, and tissue edema^6^. Fluid overload is a common complication in critically ill patients with AKI and affects approximately two-thirds of patients with AKI requiring RRT^7,8^. Fluid overload has also been reported to be associated with increased mortality and reduced renal recovery in patients with AKI^9–15^. Patients with sepsis-induced AKI (SIAKI) are particularly at risk of fluid overload because early aggressive fluid resuscitation has been the mainstay of standard care for patients with severe sepsis and septic shock since the results of early goal-directed therapy (EGDT) have been published^16^.

Though conservative fluid management and diuretics have been generally proposed to avoid fluid overload, continuous RRT (CRRT) has been considered an essential strategy for fluid management in critically ill patients with AKI. Previous studies also showed that fluid removal using CRRT was associated with improved outcomes in critically ill patients with AKI^7,8,17^.

We previously reported that fluid overload was associated with increased mortality in critically ill patients with AKI receiving CRRT^13^. In that study, subgroup analysis showed that the adverse effect of fluid overload on survival was evident in patients with SIAKI^13^. However, our previous study had a limitation owing to the small number of patients with SIAKI (n = 201), and studies on the effect of fluid overload on mortality in critically ill patients with SIAKI receiving CRRT have been scarce. Based on our previous study, the present study was conducted with the following aims. Aim 1: to find the optimal cutoff value of the degree of fluid overload for predicting mortality in critically ill patients with SIAKI receiving CRRT. Aim 2: to find an independent association between fluid overload and mortality in these patients. Aim 3: to determine whether minimizing fluid overload through CRRT is associated with decreased mortality.

## Methods

### Study population

We conducted a single-center, retrospective cohort study of patients admitted to the ICU at Pusan National University Yangsan Hospital between 2015 and 2020. A total of 647 adult patients (age ≥ 18 years) with sepsis and AKI who underwent CRRT were initially recruited. The exclusion criteria were as follows: (a) end-stage renal disease on chronic dialysis or history of kidney transplantation; (b) missing data related to admission weight or fluid balance, and (c) mortality within 24 h of CRRT initiation. Finally, a total of 543 patients were examined (Supplementary Fig. S1). All research and data collection processes were conducted in accordance with the Declaration of Helsinki and current ethical guidelines. The study protocol was approved by the hospital’s institutional review board (IRB) (Pusan National University Yangsan Hospital Review Board, IRB No. 05–2021-140). All research and data collection processes were conducted in accordance with the Declaration of Helsinki and current ethical guidelines. The Institutional Review Board of Pusan National University Yangsan Hospital waived the need for informed consent due to the retrospective nature of the analysis, which only used the information available from anonymized medical charts and records.

### Data collection

We reviewed electronic medical records. Demographic and clinical data at ICU admission, including age, sex, body weight, comorbid diseases (chronic kidney disease [CKD], hypertension, diabetes, chronic obstructive pulmonary disease [COPD], liver cirrhosis, congestive heart failure, solid cancer, and hematologic cancer), infection source (respiratory, gastrointestinal, urinary tract, and soft tissue), severity of illness (Sequential Organ Failure Assessment [SOFA] score), Acute Physiology and Chronic Health Evaluation II (APACHE II), vasopressor use, and ventilator dependency), mean arterial pressure, fever, heart rate, and oliguria (< 0.5 mL/kg per hour for 6 h before CRRT initiation), were collected. Blood examinations were performed upon ICU admission and included creatinine, blood urea nitrogen, potassium, sodium, leukocyte count, hemoglobin, platelet count, total bilirubin, albumin, prothrombin time (international normalized ratio) (PT [INR]), C-reactive protein, procalcitonin, and lactate. Finally, the intervals between AKI diagnosis and CRRT initiation, CRRT duration, and fluid overload status were investigated.

### CRRT protocol

The primary indications for CRRT initiation were medically intractable volume overload, electrolyte imbalance, metabolic acidosis, oliguria with progressive azotemia, and hemodynamic instability in patients with sepsis and AKI. Decisions regarding when to initiate or terminate CRRT and the CRRT setting (target clearance, blood flow, dialysate/replacement fluid rate, and anticoagulation) were made through consultations and discussions with attending nephrologists. All patients received continuous veno-venous hemodiafiltration using Prisma or Prismaflex (Baxter, IL, USA) with an AN-69 polyacrylonitrile membrane dialyzer. A venous catheter for CRRT was inserted into the internal jugular or femoral vein. CRRT was initiated with blood flow, which was gradually increased to 150 mL/min. A CRRT dose of 35–40 mL/kg per hour was prescribed to ensure a delivered CRRT dose of ≥ 35 mL/kg per hour.

### Definition and study outcome

Sepsis was defined according to the American College of Chest Physicians/Society of Critical Care Medicine consensus conference criteria^18^. If patients had a proven or strongly suspected bacterial infection and had at least two of the systemic inflammatory response syndrome criteria (body temperature > 38 °C or < 36 °C, heart rate > 90 bpm, respiratory rate > 20 breaths/min, PaCO_2_ < 32 mmHg or use of mechanical ventilation, white cell count > 12,000/mm^3^ or < 4000/mm^3^, or immature neutrophils > 10%), sepsis was diagnosed. AKI diagnosis was based on the Kidney Disease: Improving Global Outcomes (KDIGO) clinical practice guidelines for AKI (increase in serum creatinine ≥ 0.3 mg/dL within 48 h, increase in serum creatinine ≥ 1.5-times the baseline value, or urine volume < 0.5/kg/h for 6 h)^19^. The primary outcome was the best cutoff value of fluid overload in predicting the 28-day mortality after ICU admission in the study population. The secondary outcome was a comparison of the 28-day mortality between the groups determined according to the best cutoff value of fluid overload.

### Fluid status assessment

All available input (intravenous fluid, blood product, total parenteral nutrition, or enteral feeding) and output (urine, drain, nasogastric tube, and stool volumes) data starting from AKI diagnosis to ICU discharge were used to assess fluid overload. The degree of cumulative fluid overload in relation to body weight was expressed as percent fluid overload (%FO), which was calculated using the following formula: ∑ daily (fluid intake in L − total output in L)/baseline body weight in kg) × 100^12^. Baseline body weight was measured upon ICU admission. Based on our previous study^13^, %FO was further subdivided into %FO from AKI diagnosis to CRRT initiation (%FOpreCRRT) and %FO from CRRT initiation to ICU discharge (%FOpostCRRT). Finally, total fluid overload (%FOtotal) from AKI diagnosis to ICU discharge was defined as %FOpreCRRT + %FOpostCRRT.

### Statistical analysis

Continuous variables are expressed as medians with interquartile ranges and were compared using the Mann–Whitney or Kruskal–Wallis test. Categorical variables are expressed as numbers with percentages and were compared using the chi-square test. Receiver operating characteristic (ROC) curve analysis was employed to assess the area under the curve (AUC), and the Youden index was used to determine the best cutoff value of %FOpreCRRT and %FOtotal for predicting the 28-day mortality. Kaplan–Meier analysis was performed within 28 days of ICU admission and compared the survival curve between the groups, which was stratified by the best cutoff value of %FOpreCRRT and %FOtotal, using the log-rank test. To determine the independent risk factors for the 28-day mortality after ICU admission, univariable and multivariable Cox proportional hazards were used, and results are presented as hazard ratios (HR) and 95% confidence intervals (CIs). Significant variables were identified through univariable analysis (*P* < 0.1), and clinically important variables were considered in the multivariable analysis. Of the significant variables in the univariable analysis, variables (mean arterial pressure, platelet count, pH, and serum creatinine) included in SOFA or APACHE II scores were excluded from multivariable analysis to avoid redundant analysis. Instead, the SOFA and APACHE II scores were considered in the final multivariable analysis on behalf of those variables. Statistical significance was set at p < 0.05. All analyses were performed using SPSS version 26.0 (SPSS, Inc., Chicago, IL, USA) and MedCalc Statistical Software version 19.4.1 (MedCalc Software, Ostend, Belgium).

## Results

### Baseline characteristics at ICU admission

A total of 543 patients with sepsis and AKI underwent CRRT. The baseline characteristics of the study population at ICU admission stratified by the 28-day mortality are presented in Table 1. The median age of the study population was 70 years, and 325 patients were men. The most common infection source in the study population was the respiratory tract, followed by the gastrointestinal tract, urinary tract, soft tissue, and unknown. Of the 543 patients, 275 died within 28 days after ICU admission. Regarding demographics, there were no significant differences in age, sex, or body weight between survivors and non-survivors. In terms of comorbid diseases, non-survivors had a higher prevalence of COPD, liver cirrhosis, and congestive heart failure than survivors. There were no significant differences in infection sources between survivors and non-survivors. Regarding the severity of illness, non-survivors had higher SOFA and APACHE II scores and prevalence of vasopressor use and ventilator dependency than survivors. Concerning findings on ICU admission, non-survivors had lower mean arterial pressure, platelet count, and pH than survivors. Non-survivors were also likely to have a higher prevalence of oliguria, along with elevated prothrombin time, procalcitonin, and lactate levels. The interval between AKI diagnosis and CRRT initiation in non-survivors was significantly higher than that in survivors. The CRRT duration in non-survivors was significantly longer than that in survivors. There were no significant differences in the prescribed CRRT doses between the two groups. In terms of fluid overload status, non-survivors had a higher %FOpreCRRT, %FOpostCRRT, and %FOtotal than survivors.

**Table 1:** Baseline characteristics stratified by mortality at 28 days after ICU admission (n = 543).

Variables	Total (n = 543)	Survivors (n = 275)	Non-survivors (n = 268)	*P*
Demographics				
Age (years)	70 (59–79)	67 (57–75)	73 (63–82)	< 0.001
Sex, male	325 (59.9%)	163 (59.3%)	162 (60.4%)	0.780
Baseline body weight (kg)	64 (56–74)	65 (56–74)	64 (57–74)	0.838
Comorbid disease, n (%)				
Chronic kidney disease	122 (22.5%)	28 (21.1%)	64 (23.9%)	0.436
Hypertension	290 (53.4%)	142 (51.6%)	148 (55.2%)	0.402
Diabetes mellitus	200 (36.8%)	98 (35.6%)	102 (38.1%)	0.558
COPD	76 (14.0%)	29 (10.5%)	47 (17.5%)	0.019
Liver cirrhosis	91 (16.8%)	33 (12.0%)	58 (21.6%)	0.003
Congestive heart failure	81 (14.9%)	29 (10.5%)	51 (19.0%)	0.008
Solid cancer	125 (23.0%)	61 (22.2%)	64 (23.9%)	0.638
Hematologic cancer	36 (6.6%)	18 (6.5%)	18 (6.7%)	0.936
Infection source, n (%)				
Respiratory	203 (37.4%)	100 (36.4%)	103 (38.4%)	0.618
Gastrointestinal	177 (32.6%)	85 (30.9%)	92 (34.3%)	0.395
Urinary tract	128 (23.6%)	73 (26.5%)	55 (20.5%)	0.098
Soft tissue	21 (3.9%)	9 (3.3%)	12 (4.5%)	0.467
Unknown	14 (2.6%)	8 (2.9%)	6 (2.2%)	0.622
Severity of illness				
SOFA score	14 (10–18)	11 (7–15)	18 (14–20)	< 0.001
APACHE II score	29 (24–33)	25 (21–30)	32 (28–36)	< 0.001
Vasopressors use, n (%)	295 (54.3%)	100 (36.4%)	195 (72.8%)	< 0.001
Ventilator dependency, n (%)	299 (55.1%)	90 (32.7%)	209 (78.0%)	< 0.001
Findings at ICU admission				
Mean arterial pressure (mmHg)	73 (60–87)	80 (69–93)	64 (53–79)	< 0.001
Fever or hypothermia, n (%)	150 (27.6%)	69 (25.1%)	81 (30.2%)	0.181
Heart rate (beat/minute)	99 (80–119)	98 (79–117)	99 (82–119)	0.348
Oliguria for 6 h before CRRT (< 0.5 ml/kg per hour), n (%)	258 (47.5%)	74 (26.9%)	184 (68.7%)	< 0.001
Creatinine (mg/dl)	2.7 (2.4–3.7)	2.8 (2.5–3.6)	2.7 (2.3–4.0)	0.581
Blood urea nitrogen (mg/dl)	50.9 (41.2–70.1)	50.0 (41.4–69.8)	51.1 (40.6–70.2)	0.778
Sodium (mEq/l)	135 (129–141)	135 (129–141)	134 (130–140)	0.460
Potassium (mEq/l)	4.7 (4.1–5.4)	4.6 (4.1–5.3)	4.8 (4.1–5.4)	0.328
Leukocyte count (1000/mm^ 3^)	13.5 (10.2–18.7)	13.5 (10.5–18.6)	13.5 (9.2–19.2)	0.596
Hemoglobin (g/dl)	9.2 (7.8–11.0)	9.0 (7.9–10.9)	9.4 (7.7–11.1)	0.765
Platelet count (1000/mm^3^)	91 (60–140)	112 (71–157)	78 (52–118)	< 0.001
Total bilirubin (mg/dl)	1.7 (0.5–3.9)	1.7 (0.5–3.9)	1.8 (0.5–3.9)	0.746
Albumin (g/dl)	2.6 (2.2–3.0)	2.6 (2.2–3.0)	2.6 (2.3–3.1)	0.297
PT(INR)	1.6 (1.4–2.0)	1.6 (1.3–2.0)	1.7 (1.5–2.1)	< 0.001
CRP (mg/dl)	12.6 (7.4–25.8)	11.5 (7.2–25.7)	14.1 (7.6–26.0)	0.358
Procalcitonin (ng/ml)	11.9 (6.0–20.4)	9.7 (4.7–16.5)	15.0 (8.3–23.8)	< 0.001
pH	7.30 (7.22–7.37)	7.32 (7.26–7.39)	7.26 (7.18–7.34)	< 0.001
Lactate (mmol/l)	6.0 (2.4–10.4)	3.3 (1.9–7.3)	7.8 (4.8–11.9)	< 0.001
CRRT				
Interval time from AKI diagnosis to CRRT initiation (days)	1.4 (0.4–2.4)	0.5 (0.3–1.5)	2.1 (1.3–2.9)	< 0.001
CRRT duration (days)	5.3 (3.3–8.9)	4.9 (2.5–8.8)	5.8 (4.0–9.2)	< 0.001
Prescribed CRRT dose (ml/kg/hr)	37.6 (35.3–39.9)	37.6 (35.4–39.8)	37.6 (35.0–39.9)	0.667
Fluid overload status^a^				
%FOpreCRRT (%)	4.7 (3.4–7.5)	3.7 (2.7–4.7)	7.2 (4.5–10.9)	< 0.001
%FOpostCRRT (%)	2.2 (-1.5–5.8)	1.0 (-3.1–3.3)	4.6 (0.2–9.1)	< 0.001
%FOtotal (%)^c^	8.3 (4.3–13.4)	4.9 (-0.3–8.0)	12.5 (9.8–17.9)	< 0.001

Values are expressed as median (interquartile range) or percentage (%).

AKI, acute kidney injury; APACHE II, Acute Physiology and Chronic Health Evaluation II; COPD, chronic obstructive pulmonary disease; CRP, C-reactive protein; CRRT, continuous renal replacement therapy; ICU, intensive care unit; PT (INR), prothrombin time (international normalized ratio); SOFA, Sequential Organ Failure Assessment.

^a^%FOpreCRRT was defined as the percentage of fluid overload relative to baseline body weight from AKI diagnosis to ICU initiation. %FOpostCRRT was defined as the percentage of fluid overload relative to baseline body weight from CRRT initiation to ICU discharge. %FOtotal was defined as the sum of %FOpreCRRT and %FOpostCRRT.

### Performance of fluid overload status for predicting the 28-day mortality

**Figure 1:**
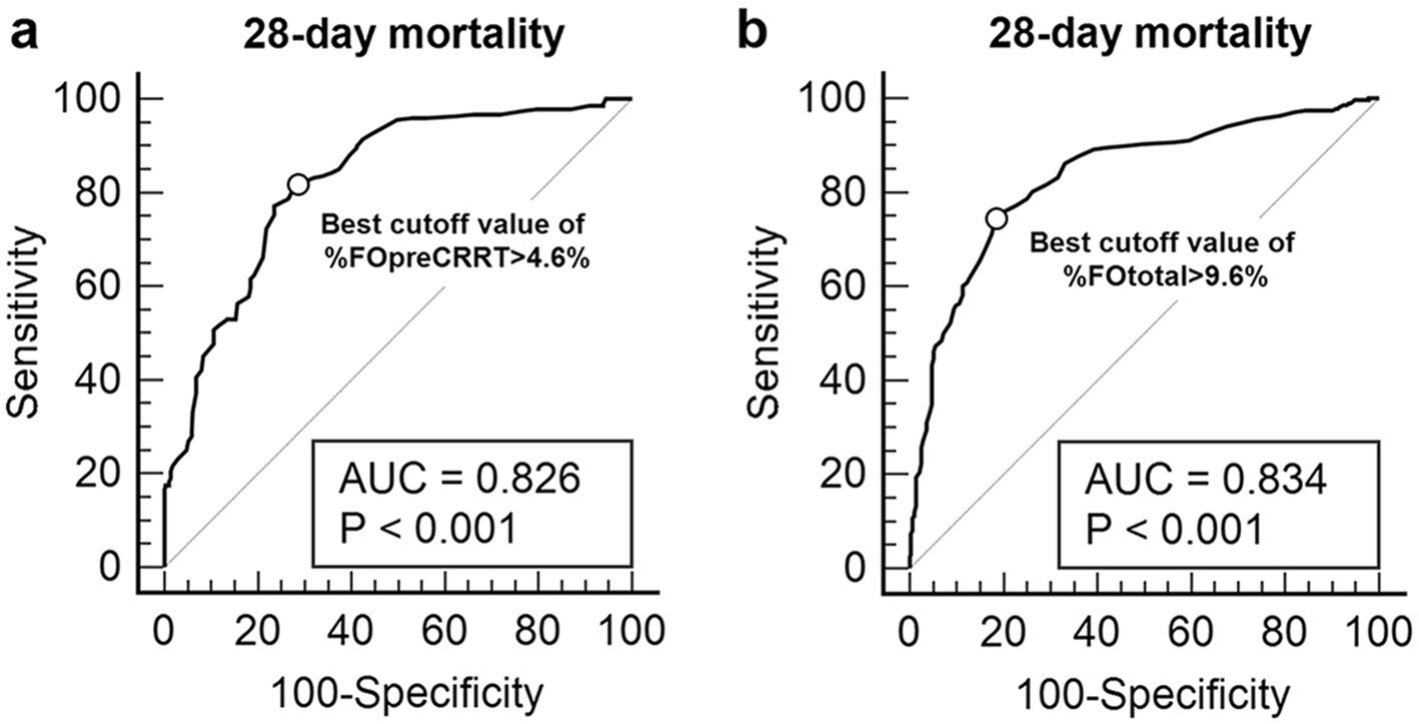
Receiver-operating characteristic curves of %FOpreCRRT (**a**) and %FOtotal (**b**) for predicting the 28-day mortality in the study population (n = 543). The AUCs were 0.826 (95% CI: 0.791–0.857, *P* < 0.001) for %FOpreCRRT and 0.834 (95% CI: 0.800–0.864, *P* < 0.001) for %FOtotal. The best cutoff value of %FOpreCRRT was > 4.6%, with an associated sensitivity of 82.5% and specificity of 73.0%. The best cutoff of %FOtotal was > 9.6%, with an associated sensitivity of 71.6% and specificity of 70.8%. %FOpreCRRT was defined as the percentage of fluid overload relative to baseline body weight from AKI diagnosis to ICU initiation. %FOpostCRRT was defined as the percentage of fluid overload relative to baseline body weight from CRRT initiation to ICU discharge. %FOtotal was defined as the sum of %FOpreCRRT and %FOpostCRRT. AKI, acute kidney injury; AUC, area under the curve; CI, confidence interval; CRRT, continuous renal replacement therapy; ICU, intensive care unit.

**Figure 2:**
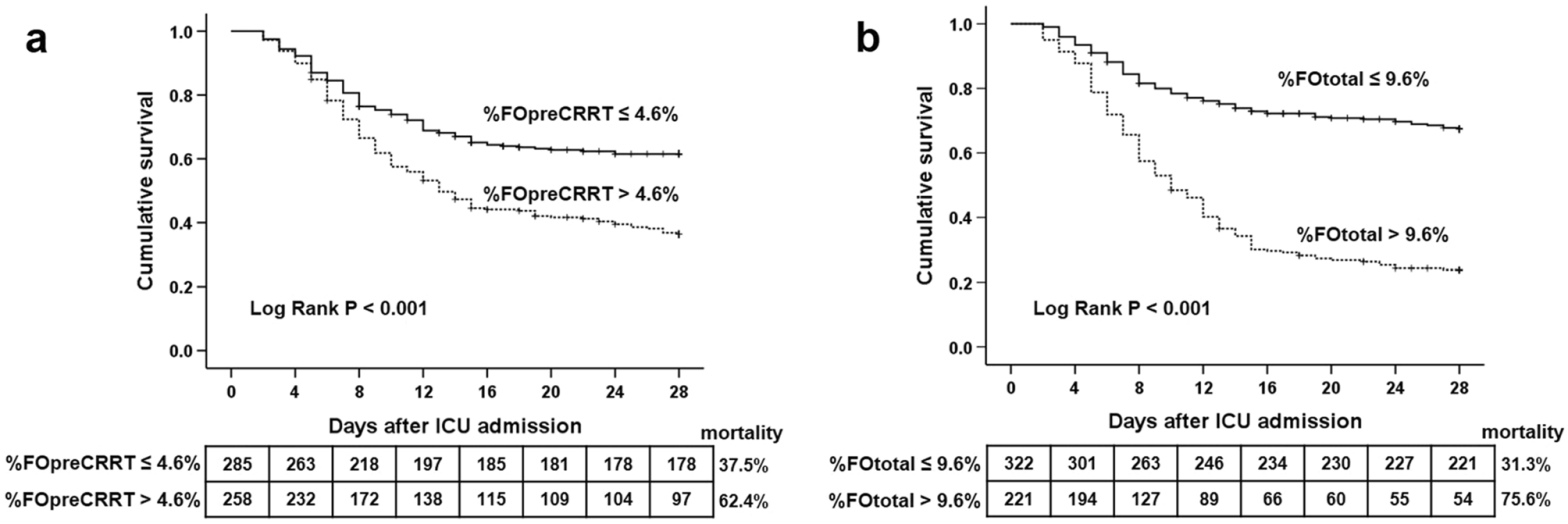
Kaplan–Meier survival estimate according to the best cutoff value of %FOpreCRRT (**a**) and %FOtotal (**b**) for 28-day mortality. There was a significant difference in survival among patients with %FOpreCRRT ≤ 4.6% or > 4.6% (28-day mortality, 37.5% vs. 62.4%, *P* < 0.001). There was also a significant difference in survival among patients with %FOtotal ≤ 9.6% or > 9.6% (28-day mortality, 31.3% vs. 75.6%, *P* < 0.001). %FOpreCRRT was defined as the percentage of fluid overload relative to baseline body weight from AKI diagnosis to ICU initiation. %FOpostCRRT was defined as the percentage of fluid overload relative to baseline body weight from CRRT initiation to ICU discharge. %FOtotal was defined as the sum of %FOpreCRRT and %FOpostCRRT. CRRT, continuous renal replacement therapy, ICU; intensive care unit.

To investigate the diagnostic power of %FOpreCRRT and %FOtotal for predicting the 28-day mortality, ROC analysis was performed (Fig. 1). The AUCs were 0.826 (95% CI: 0.791–0.857, *P* < 0.001) for %FOpreCRRT and 0.834 (95% CI: 0.800–0.864, *P* < 0.001) for %FOtotal. The best cutoff value of %FOpreCRRT was > 4.6%, with an associated sensitivity of 82.5% and specificity of 73.0%. The best cutoff of %FOtotal was > 9.6%, with an associated sensitivity of 71.6% and specificity of 70.8%. Next, we divided the study subjects into those with %FOpreCRRT > 4.6% and those with %FOpreCRRT ≤ 4.6%, or those with %FOtotal > 9.6% and those with %FOtotal ≤ 9.6% according to the best cutoff value of %FOpreCRRT and %FOtotal for the 28-day mortality. Patients with %FOpreCRRT > 4.6% showed a significant decrease in survival after ICU admission compared with those with %FOpreCRRT ≤ 4.6% (28-day mortality: 37.5% vs. 62.4%, *P* < 0.001). There was also a significant difference in survival between patients with %FOtotal > 9.6% and those with %FOtotal ≤ 9.6% (28-day mortality: 31.3% vs. 75.6%, *P* < 0.001) (Fig. 2).

### Association between %FOpreCRRT or %FOtotal and the 28-day mortality

Table 2 shows the variables found to be associated with the 28-day mortality in the univariable Cox regression analysis. %FOpreCRRT > 4.6% (vs. ≤ 4.6%, HR: 1.90, 95% CI: 1.49–2.43, *P* < 0.001) and %FOtotal > 9.6% (vs. ≤ 9.6%, HR: 3.37; 95% CI: 2.63–4.33, *P* < 0.001) were significant predictors for the 28-day mortality. Furthermore, age, COPD, liver cirrhosis, congestive heart failure, SOFA score, APACHE II score, vasopressor use, ventilator use, mean arterial pressure, oliguria, platelet count, PT (INR), procalcitonin, pH, lactate, and time from AKI diagnosis to CRRT initiation were significant predictors of the 28-day mortality. Table 3 shows the results of the multivariable Cox proportional hazards analysis for the 28-day mortality after ICU admission in the study population. %FOpreCRRT > 4.6% (vs. ≤ 4.6%, HR: 1.42; 95% CI: 1.10–1.83, *P* < 0.001) and %FOtotal > 9.6% (vs. ≤ 9.6%, HR: 1.95, 95% CI: 1.49–2.56, *P* < 0.001) were significant predictors for the 28-day mortality. In addition, SOFA score (per 1 point increment, HR: 1.11, 95% CI: 1.07–1.14, *P* < 0.001), APACHE II score (per 1 point increment; HR: 1.05, 95% CI: 1.03–1.07, *P* < 0.001), oliguria before CRRT (HR: 1.48, 95% CI: 1.10–1.98, *P* = 0.009), lactate (per 1.0 mmol/L increment, HR: 1.05, 95% CI: 1.02–1.08, *P* = 0.003), and time from AKI diagnosis to CRRT initiation (per 1 day increment, HR: 1.11, 95% CI: 1.10–1.83, *P* = 0.047) were significantly associated with the 28-day mortality.

**Table 2:** Univariable analysis for the 28-day mortality after ICU admission for all subjects (n = 543).

Variables	HR	95% CI	*P*-value
Demographics			
Age (per 1 year increment)	1.02	1.01–1.03	< 0.001
Sex, male	1.01	0.79–1.29	0.921
Baseline body weight (per 1.0 kg increment)	1.00	0.99–1.01	0.770
Comorbid disease			
Chronic kidney disease	1.07	0.80–1.41	0.637
Hypertension	1.07	0.84–1.37	0.551
Diabetes mellitus	1.05	0.82–1.35	0.676
Chronic obstructive pulmonary disease	1.46	1.06–2.00	0.018
Liver cirrhosis	1.46	1.09–1.96	0.010
Congestive heart failure	1.50	1.10–2.03	0.010
Solid cancer	1.05	0.79–1.40	0.741
Hematologic cancer	1.06	0.66–1.72	0.802
Infection source			
Respiratory	1.07	0.84–1.38	0.565
Gastrointestinal	1.09	0.84–1.40	0.521
Urinary tract	0.79	0.59–1.06	0.118
Soft tissue	1.34	0.75–2.40	0.338
Unknown	0.81	0.36–1.82	0.610
Severity of illness			
SOFA score (per 1 point increment)	1.17	1.14–1.21	< 0.001
APACHE II score (per 1 point increment)	1.12	1.10–1.15	< 0.001
Vasopressors use	2.99	2.28–3.92	< 0.001
Ventilator dependency	4.27	3.19–5.71	< 0.001
Findings at ICU admission			
Mean arterial pressure (per 1.0 mmHg increment)	0.96	0.95–0.97	< 0.001
Fever or hypothermia	1.19	0.92–1.54	0.195
Heart rate (per 1.0 beat/minute increment)	1.00	1.00–1.01	0.311
Oliguria for 6 h before CRRT (< 0.5 ml/kg per hour)	4.00	2.62–4.41	< 0.001
Serum creatinine (per 1.0 mg/dl increment)	1.05	0.96–1.16	0.274
Serum blood urea nitrogen (per 1.0 mg/dl increment)	1.00	1.00–1.00	0.747
Sodium (per 1.0 mEq/l increment)	1.00	1.00–1.01	0.658
Potassium (per 1.0 mEq/l increment)	1.03	0.91–1.17	0.630
Leukocyte count (per 1000/mm^3^ increment)	1.00	0.99–1.02	0.851
Hemoglobin (per 1.0 g/dl increment)	0.99	0.93–1.04	0.646
Platelet count (per 1000/mm^3^ increment)	1.00	0.99–1.00	< 0.001
Total bilirubin (per 1.0 mg/dl increment)	1.02	0.97–1.07	0.422
Albumin (per 1.0 g/dl increment)	1.14	0.91–1.43	0.244
PT(INR) (per 1.0 increment)	1.25	1.05–1.50	0.014
CRP (per 1.0 mg/dl increment)	1.00	0.99–1.01	0.631
Procalcitonin (per 1.0 ng/ml increment)	1.02	1.01–1.03	< 0.001
pH (per 0.1 increment)	0.63	0.25–1.60	< 0.001
Lactate (per 1.0 mmol/l increment)	1.13	1.10–1.15	< 0.001
CRRT			
Interval time from AKI diagnosis to CRRT initiation (per 1 day increment)	1.56	1.44–1.69	< 0.001
CRRT duration (per 1 day increment)	0.99	0.96–1.01	0.283
Prescribed CRRT dose (per 1 ml/kg/hr increment)	0.99	0.95–1.04	0.732
Fluid overload status^a^			
% FOpreCRRT > 4.6% (vs. ≤ 4.6%)	1.90	1.49–2.43	< 0.001
% FOtotal > 9.6% (vs. ≤ 9.6%)	3.37	2.63–4.33	< 0.001

AKI, acute kidney injury; APACHE II, Acute Physiology and Chronic Health Evaluation II; COPD, chronic obstructive pulmonary disease; CRP, C-reactive protein; CRRT, continuous renal replacement therapy; ICU, intensive care unit; PT (INR), prothrombin time (international normalized ratio); SOFA, Sequential Organ Failure Assessment.

^a^%FOpreCRRT was defined as the percentage of fluid overload relative to baseline body weight from AKI diagnosis to ICU initiation. %FOpostCRRT was defined as the percentage of fluid overload relative to baseline body weight from CRRT initiation to ICU discharge. %FOtotal was defined as the sum of %FOpreCRRT and %FOpostCRRT.

**Table 3:** Multivariable Cox proportional hazards analysis for the 28-day mortality after ICU admission for all subjects (n = 543).

Variables	Adjusted HR	95% CI	*P*-value
Age (per 1 year increment)	1.00	0.99–1.01	0.889
Sex, male	1.03	0.79–1.33	0.844
COPD	1.08	0.81–1.47	0.660
Liver cirrhosis	1.09	0.81–1.47	0.577
Congestive heart failure	1.26	0.91–1.73	0.171
SOFA score (per 1 point increment)	1.11	1.07–1.14	< 0.001
APACHE II score (per 1 point increment)	1.05	1.03–1.07	< 0.001
Oliguria for 6 h before CRRT (< 0.5 ml/kg per hour)	1.48	1.10–1.98	0.009
PT(INR) (per 1.0 increment)	0.90	0.72–1.12	0.321
Procalcitonin (per 1.0 ng/ml increment)	1.00	0.99–1.01	0.920
Lactate (per 1.0 mmol/l increment)	1.05	1.02–1.08	0.003
Interval time from AKI diagnosis to CRRT initiation (per 1 day increment)	1.11	1.10–1.83	0.047
Fluid overload status^a^			
% FOpreCRRT > 4.6% (vs. ≤ 4.6%)	1.42	1.10–1.83	0.007
% FOtotal > 9.6% (vs. ≤ 9.6%)	1.95	1.49–2.56	< 0.001

AKI, acute kidney injury; APACHE II, Acute Physiology and Chronic Health Evaluation II; COPD, chronic obstructive pulmonary disease; CRP, C-reactive protein; CRRT, continuous renal replacement therapy; ICU, intensive care unit; PT (INR), prothrombin time (international normalized ratio); SOFA, Sequential Organ Failure Assessment.

^a^%FOpreCRRT was defined as the percentage of fluid overload relative to baseline body weight from AKI diagnosis to ICU initiation. %FOpostCRRT was defined as the percentage of fluid overload relative to baseline body weight from CRRT initiation to ICU discharge. %FOtotal was defined as the sum of %FOpreCRRT and %FOpostCRRT.

**Figure 3:**
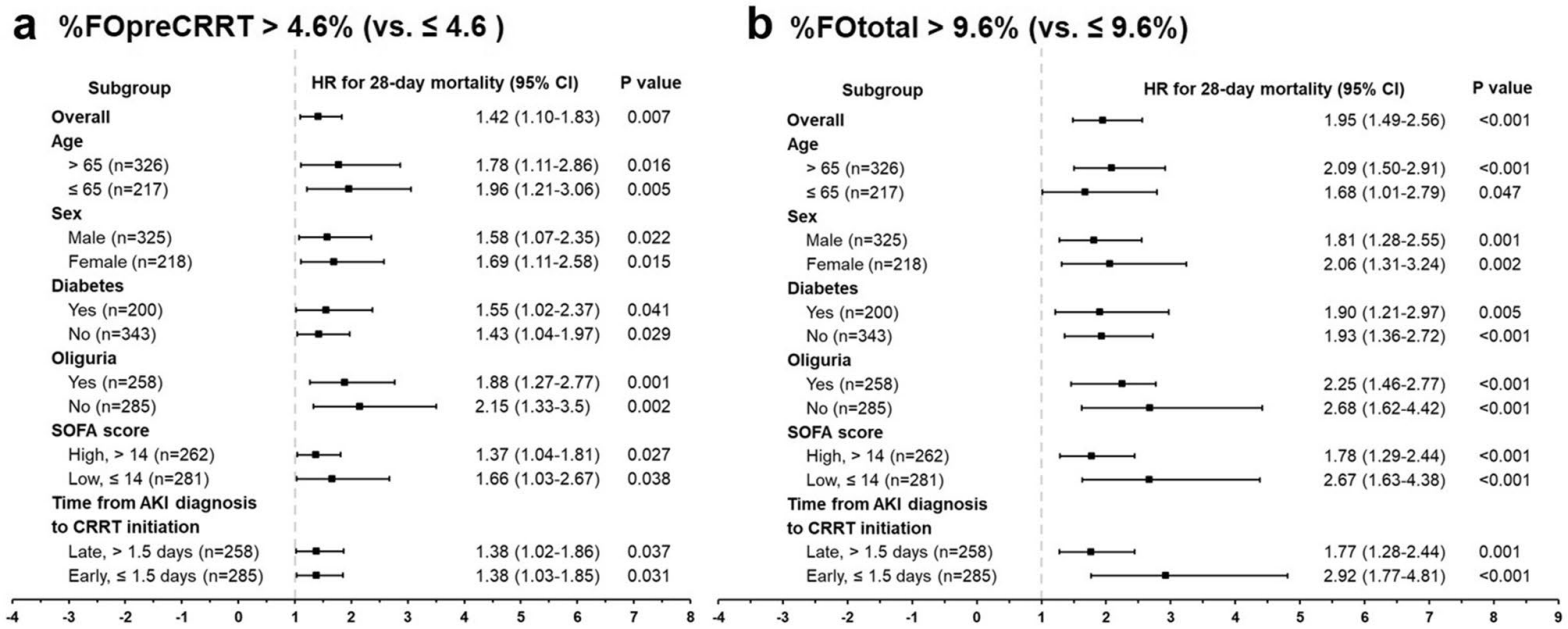
HR plots of %FOpreCRRT (**a**) and %FOtotal (**b**) for 28-day mortality according to subgroup. %FOpreCRRT > 4.6% (vs. ≤ 4.6%) and %FOtotal > 9.6% (vs. ≤ 9.6%) was associated with an increased HR for 28-day mortality in all subgroups. The HR was analyzed with a fully adjusted Cox regression model including age, sex, COPD, liver cirrhosis, CHF. SOFA score, APACHE II score, PT (INR), procalcitonin, lactate, and time from AKI diagnosis to CRRT initiation. %FOpreCRRT was defined as a percentage of fluid overload from baseline body weight from AKI diagnosis to ICU initiation. %FOpostCRRT was defined as a percentage of fluid overload from baseline body weight from CRRT initiation to ICU discharge. %FOtotal was defined as the sum of %FOpreCRRT and %FOpostCRRT. Oliguria was defined as < 0.5 mL/kg per hour of urine output for 6 h before CRRT initiation. AKI, acute kidney injury; APACHE II, Acute Physiology and Chronic Health Evaluation II; COPD, chronic obstructive pulmonary disease; CRP, C-reactive protein; CRRT, continuous renal replacement therapy; HR, hazard ratio; ICU, intensive care unit; PT (INR), prothrombin time (international normalized ratio); SOFA, sequential organ failure assessment.

Next, we analyzed the association between fluid overload and the 28-day mortality in subgroups stratified by age, sex, diabetes, oliguria, SOFA score, and time from AKI diagnosis to CRRT initiation. First, we performed ROC analysis to determine the best cutoff value of the SOFA score and time from AKI diagnosis to CRRT initiation for predicting the 28-day mortality. Best cutoff values of the SOFA score and time from AKI diagnosis to CRRT initiation were > 14 points (AUC, 0.842; P < 0.001) and > 1.5 days (AUC, 0.808; *P* < 0.001), respectively (Supplementary Fig. S2). Based on the best cutoff value, we divided the study subjects into a high SOFA group (> 14 points) or low SOFA group (≤ 14 points) and late CRRT group (> 1.5 days) or early CRRT group (≤ 1.5 days). In multivariable Cox regression analysis (Fig. 3), %FOpreCRRT > 4.6% (vs. ≤ 4.6) and %FOtotal > 9.6% (vs. ≤ 9.6%) were significant predictors of the 28-day mortality irrespective of high or low SOFA scores and early or late CRRT. Independent associations between fluid overload and the 28-day mortality were consistently observed in other subgroups, including age > 65 or ≤ 65 years, male or female sex, diabetes or no diabetes, and oliguria or no oliguria.

### Effect of minimizing fluid overload using CRRT on the 28-day mortality

All patients were also classified into four groups to assess the effect of minimizing the fluid overload using CRRT on mortality: Group 1 (n = 182, %FOpreCRRT ≤ 4.6% and %FOtotal ≤ 9.6%; no significant fluid overload before and after CRRT application, and finally, no significant total fluid overload); Group 2 (n = 140, %FOpreCRRT > 4.6% and %FOtotal ≤ 9.6%; significant fluid overload before CRRT that was then resolved by CRRT, and finally, no significant total fluid overload); Group 3 (n = 103, %FOpreCRRT ≤ 4.6% and %FOtotal > 9.6%; no significant fluid overload before CRRT, but significant total fluid overload due to aggravation of fluid overload during CRRT); and Group 4 (n = 118, %FOpreCRRT > 4.6% and %FOtotal > 9.6%; significant fluid overload before CRRT, which was not resolved by CRRT, and finally, significant total fluid overload).

**Figure 4:**
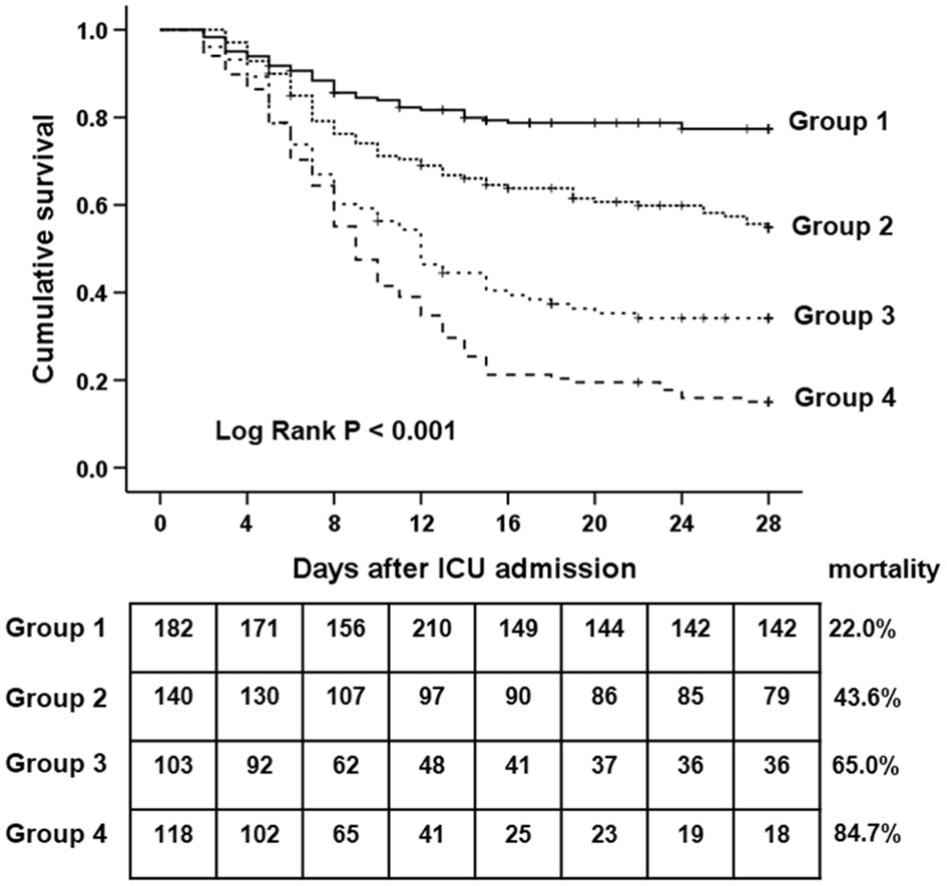
Kaplan–Meier survival estimate among the four groups categorized by the best cutoff value of %FOpreCRRT and %FOtotal for 28-day mortality. There was a significant difference in survival between the four groups. Twenty-eight-day mortality was the highest in Group 4, followed by Group 3, Group 2, and Group 1 (84.7% vs. 65.0% vs. 43.6% vs. 22.0%, *P* < 0.001). Group 1 (n = 182): %FOpreCRRT ≤ 4.6% and %FOtotal ≤ 9.6%; Group 2 (n = 140): %FOpreCRRT > 4.6% and %FOtotal ≤ 9.6%; Group 3 (n = 103): %FOpreCRRT ≤ 4.6% and %FOtotal > 9.6%; Group 4 (n = 118): %FOpreCRRT > 4.6% and %FOtotal > 9.6% %FOpreCRRT was defined as the percentage fluid overload relative to baseline body weight from AKI diagnosis to ICU initiation. %FOpostCRRT was defined as the percentage fluid overload relative to baseline body weight from CRRT initiation to ICU discharge. %FOtotal was defined as the sum of %FOpreCRRT and %FOpostCRRT. CRRT, continuous renal replacement therapy, ICU; intensive care unit.

Table 4 shows the baseline characteristics of the four subgroups. There were significant differences among the four groups with respect to age, SOFA score, APACHE II score, vasopressor use, ventilator dependency, mean arterial pressure, oliguria, platelet count, procalcitonin, pH, lactate, time from AKI diagnosis to CRRT initiation, and CRRT duration. There was a significant difference in survival between the four groups; the 28-day mortality was the highest in Group 4, followed by Group 3, Group 2, and Group 1 (84.7% vs. 65.0% vs. 43.6% vs. 22.0%, respectively; *P* < 0.001) (Fig. 4).

**Table 4:** Baseline characteristics stratified by fluid overload according to %FOpreCRRT and %FOtotal^a^ (n = 543).

Variables	Group 1 (n = 182)	Group 2 (n = 140)	Group 3 (n = 103)	Group 4 (n = 118)	*P*
Demographics					
Age (years)	66 (55–75)	71 (61–80)	70 (62–83)	73 (60–81)	< 0.001
Sex, male	141 (62.9%)	45 (53.6%)	37 (58.7%)	102 (59.3%)	0.780
Baseline body weight (kg)	65 (56–73)	64 (55–74)	65 (57–77)	64 (57–73)	0.656
Comorbid disease, n (%)					
Chronic kidney disease	52 (23.2%)	16 (19.0%)	16 (25.4%)	38(22.1%)	0.811
Hypertension	113 (50.4%)	45 (53.6%)	37 (58.7%)	95 (55.2%)	0.629
Diabetes mellitus	82 (36.6%)	31 (36.9%)	23 (36.5%)	64 (37.2%)	0.558
COPD	27 (12.1%)	9 (10.7%)	14 (22.2%)	26 (15.1%)	0.159
Liver cirrhosis	35 (15.6%)	12 (14.3%)	10 (15.9%)	34 (19.8%)	0.631
Congestive heart failure	28 (12.5%)	7 (8.3%)	14 (22.2%)	32 (18.6%)	0.039
Solid cancer	48 (21.4%)	18 (21.4%)	13 (20.6%)	46 (26.7%)	0.575
Hematologic cancer	14 (6.3%)	5 (6.0%)	7 (11.1%)	10 (5.8%)	0.504
Infection source, n (%)					
Respiratory	77 (34.4%)	31 (36.9%)	29 (46.0%)	69 (40.1%)	0.341
Gastrointestinal	70 (31.3%)	26 (31.0%)	17 (27.0%)	64 (37.4%)	0.414
Urinary tract	62 (27.7%)	23 (27.4%)	13 (20.6%)	30 (17.4%)	0.083
Soft tissue	6 (2.7%)	2 (2.4%)	6 (9.5%)	7 (4.1%)	0.079
Unknown	9 (4.0%)	2 (2.4%)	1 (1.6%)	2 (1.2%)	0.323
Severity of illness					
SOFA score	12 (7–15)	14 (10–19)	14 (12–17)	18 (15–20)	< 0.001
APACHE II score	26 (22–30)	29 (23–35)	30 (25–34)	32 (29–36)	< 0.001
Vasopressors use, n (%)	91 (36.2%)	48 (57.1%)	33 (52.4%)	133 (77.3%)	< 0.001
Ventilator dependency, n (%)	68 (30.4%)	51 (60.7%)	41 (65.1%)	139 (80.8%)	< 0.001
Findings at ICU admission					
Mean arterial pressure (mmHg)	80 (69–93)	70 (62–84)	73 (63–85)	62 (52–76)	< 0.001
Fever or hypothermia, n (%)	60 (26.8%)	22 (26.2%)	15 (23.8%)	53 (30.8%)	0.683
Heart rate (beat/minute)	97 (78–119)	99 (82–117)	99 (87–115)	99 (82–115)	0.911
Oliguria for 6 h before CRRT (< 0.5 ml/kg per hour), n (%)	68 (30.4%)	35 (41.7%)	31 (49.2%)	124 (72.1%)	< 0.001
Creatinine (mg/dl)	2.8 (2.5–3.7)	2.8 (2.4–3.7)	2.7 (2.4–3.4)	2.7 (2.3–4.2)	0.258
Blood urea nitrogen (mg/dl)	50.8 (41.4–65.8)	48.2 (41.8–70.7)	54.8 (41.0–88.8)	51.3 (40.3–70.6)	0.737
Sodium (mEq/l)	135 (129–140)	134 (129–139)	134 (130–141)	135 (130–142)	0.485
Potassium (mEq/l)	4.7 (4.1–5.4)	4.6 (4.1–5.3)	4.8 (4.1–5.4)	4.8 (4.1–5.4)	0.328
Leukocyte count (1000/mm^3^)	13.7 (10.8–18.4)	13.4 (10.2–17.9)	11.7 (8.0–17.0)	13.9 (9.7–20.5)	0.078
Hemoglobin (g/dl)	9.1 (7.9–10.9)	9.0 (7.7–11.0)	9.2 (8.0–11.0)	9.6 (7.7–11.0)	0.959
Platelet count (1000/mm^3^)	110 (71–156)	89 (63–136)	90 (67–132)	73 (51–117)	< 0.001
Total bilirubin (mg/dl)	1.9 (0.5–3.8)	1.2 (0.5–3.6)	1.5 (0.4–4.3)	2.0 (0.6–4.3)	0.263
Albumin (g/dl)	2.6 (2.2–2.9)	2.5 (2.1–3.0)	2.7 (2.4–3.2)	2.6 (2.3–3.0)	0.146
PT(INR)	1.6 (1.3–2.0)	1.6 (1.4–2.0)	1.6 (1.5–1.9)	1.7 (1.5–2.1)	< 0.001
CRP (mg/dl)	11.6 (7.0–25.6)	14.2 (8.1–26.9)	13.9 (6.9–26.9)	14.2 (7.8–25.2)	0.772
Procalcitonin (ng/ml)	9.8 (4.9–18.6)	10.7 (6.3–19.1)	12.7 (4.8–21.6)	15.2 (9.6–22.7)	< 0.001
pH	7.31 (7.25–7.38)	7.31 (7.23–7.37)	7.31 (7.21–7.37)	7.26 (7.19–7.35)	0.003
Lactate (mmol/l)	3.4 (2.0–7.7)	6.9 (2.7–10.7)	6.5 (3.2–9.9)	7.9 (4.4–11.9)	< 0.001
CRRT					
Interval time from AKI diagnosis to CRRT initiation (days)	0.5 (0.3–1.5)	1.5 (0.7–2.3)	1.6 (0.5–2.6)	2.2 (1.4–3.2)	< 0.001
CRRT duration (days)	4.6 (2.4–8.5)	5.7 (4.5–9.8)	5.8 (3.7–9.5)	5.8 (4.0–8.5)	< 0.001
Prescribed CRRT dose (ml/kg/hr)	37.7 (35.4–39.9)	37.6 (34.9–40.0)	37.6 (35.0–39.6)	37.5 (35.0–39.9)	0.872

Values are expressed as median (interquartile range) or percentage (%). Group 1 (n = 182): %FOpreCRRT ≤ 4.6% and %FOtotal ≤ 9.6%; Group 2 (n = 140): %FOpreCRRT > 4.6% and %FOtotal ≤ 9.6%; Group 3 (n = 103): %FOpreCRRT ≤ 4.6% and %FOtotal > 9.6%; Group 4 (n = 118), %FOpreCRRT > 4.6% and %FOtotal > 9.6%.

%FOpreCRRT was defined as the percentage of fluid overload relative to baseline body weight from AKI diagnosis to ICU initiation. %FOpostCRRT was defined as the percentage of fluid overload relative to baseline body weight from CRRT initiation to ICU discharge. %FOtotal was defined as the sum of %FOpreCRRT and %FOpostCRRT.

AKI, acute kidney injury; APACHE II, Acute Physiology and Chronic Health Evaluation II; COPD, chronic obstructive pulmonary disease; CRP, C-reactive protein; CRRT, continuous renal replacement therapy; ICU, intensive care unit; PT (INR), prothrombin time (international normalized ratio); SOFA, Sequential Organ Failure Assessment.

We further analyzed the 28-day mortality according to the four groups using the multivariable Cox proportional hazards analysis (Table 5). In the univariable analysis (model 1), Group 4 (HR: 6.06, 95% CI: 4.19–8.78, *P* < 0.001), Group 3 (HR: 4.01, 95% CI: 2.71–5.95, *P* < 0.001), and Group 2 (HR: 2.18, 95% CI: 1.46–3.25, *P* < 0.001) had a significantly higher risk of the 28-day mortality than Group 1. These associations were significant after adjustment for sex, age, SOFA score, APACHE II score, oliguria, and time from AKI diagnosis to CRRT initiation (model 2) (Group 4 [HR: 3.00, 95% CI: 2.03–4.45, *P* < 0.001], Group 3 [HR: 2.12, 95% CI: 1.40–3.20, *P* < 0.001], and Group 2 [HR: 1.59, 95% CI: 1.06–2.39, *P* = 0.025] compared with Group 1). These associations also remained significant after further adjustment for liver cirrhosis, COPD, congestive heart failure, prothrombin time, procalcitonin, and lactate (model 3) (Group 4 [HR: 2.90, 95% CI: 1.95–4.32, *P* < 0.001], Group 3 [HR: 2.11, 95% CI: 1.39–3.21, *P* < 0.001], and Group 2 [HR: 1.52, 95% CI: 1.01–2.30, *P* = 0.046] compared with Group 1).

**Table 5:** Multivariable Cox proportional hazards analysis for the 28-day mortality based on %FOpreCRRT and FOtotal (n = 543).

Fluid overload status^a^	Model 1		Model 2		Model 3	
	HR (95% CI)	*P*	HR (95% CI)	*P*	HR (95% CI)	*P*
Group 1	1.00 (Reference)		1.00 (Reference)		1.00 (Reference)	
Group 2	2.18 (1.46–3.25)	< 0.001	1.59 (1.06–2.39)	0.025	1.52 (1.01–2.30)	0.046
Group 3	4.01 (2.71–5.95)	< 0.001	2.12 (1.40–3.20)	< 0.001	2.11 (1.39–3.21)	< 0.001
Group 4	6.06 (4.19–8.78)	< 0.001	3.00 (2.03–4.45)	< 0.001	2.90 (1.95–4.32)	< 0.001

Model 1: Unadjusted hazard ratio. Model 2: adjusted for age, sex, SOFA score, APACHE II score, oliguria for 6 h before CRRT, and interval between AKI diagnosis and CRRT initiation. Model 3: Model 2 plus liver cirrhosis, COPD, congestive heart failure, prothrombin time, procalcitonin, and lactate levels.

Group 1 (n = 182): %FOpreCRRT ≤ 4.6% and %FOtotal ≤ 9.6%; Group 2 (n = 140): %FOpreCRRT > 4.6% and %FOtotal ≤ 9.6%; Group 3 (n = 103): %FOpreCRRT ≤ 4.6% and %FOtotal > 9.6%; Group 4 (n = 118): %FOpreCRRT > 4.6% and %FOtotal > 9.6%.

%FOpreCRRT was defined as the percentage of fluid overload relative to baseline body weight from AKI diagnosis to ICU initiation. %FOpostCRRT was defined as the percentage of fluid overload relative to baseline body weight from CRRT initiation to ICU discharge. %FOtotal was defined as the sum of %FOpreCRRT and %FOpostCRRT.

AKI, acute kidney injury; APACHE II, Acute Physiology and Chronic Health Evaluation II; COPD, chronic obstructive pulmonary disease; CRP, C-reactive protein; CRRT, continuous renal replacement therapy; ICU, intensive care unit; PT (INR), prothrombin time (international normalized ratio); SOFA, Sequential Organ Failure Assessment.

## Discussion

Sepsis, especially severe sepsis and septic shock, is a condition of hypoperfusion in the setting of infection and is associated with high mortality and morbidity, including end-organ dysfunction and failure^20^. In the management of sepsis, EGDT has been adopted either completely or at least in components in many ICUs worldwide since it showed a survival benefit in patients with severe sepsis and septic shock^16^, and the guidelines recommend the EGDT protocol^21^. EGDT requires initial aggressive fluid resuscitation to avoid hypovolemia and its associated complications, including hypotension, kidney injury, and multiorgan failure^20^. However, beyond the threshold needed for acute resuscitation, excessive fluid accumulation leads to harmful consequences, such as hypertension, pulmonary edema, respiratory failure, and increased cardiac demand^20^. Indeed, previous studies comparing EGDT and usual care with conservative fluid administration in patients with septic shock demonstrated that EGDT did not reduce all-cause mortality when compared with usual care^22–24^. Furthermore, some studies reported that fluid overload was associated with increased mortality in patients with severe sepsis and septic shock and recommended a more cautious approach to fluid resuscitation^25–27^. However, several studies reported no difference in mortality among the patients who received restricted fluid therapy and those who received liberal fluid therapy^28–31^. A meta-analysis including the 637 patients with sepsis found no statistically significant difference between lower vs. higher fluid volume in all-cause mortality^30^. Furthermore, a recent randomized controlled study including 1,554 patients showed that restricted fluid therapy did not result in fewer deaths at 90 days than standard fluid therapy among patients with septic shock in ICU^31^. Thus, optimal management of fluid balance in patients with severe sepsis or septic shock still needs to be investigated.

SIAKI is the first cause of AKI in the ICU, and 15–20% of patients with SIAKI require RRT^32^. Though SIAKI is a multifactorial syndrome with inflammatory, nephrotoxic, and ischemic insults^33^, acute tubular necrosis due to decreased kidney perfusion is known as the main pathophysiological factor of SIAKI. Thus, in line with management for patients with severe sepsis and septic shock, fluid therapy has been an essential component of care for patients with SIAKI to prevent further ischemic kidney damage. However, recent studies suggest that, in addition to decreased renal perfusion, renal inflammation and microvascular dysfunction play pivotal roles in SIAKI^33,34^. Inflammatory mediators derived from bacterial or immune cells are filtered in the glomerulus, enter the tubular space, and can subsequently injure the tubular cells by binding to their respective receptors^34^. Furthermore, cytokines, damage-associated molecular patterns (DAMPs), and pathogen-associated molecular patterns (PAMPs) are released from extravasated leukocytes and can activate tubular cells from the interstitial side. The activation of cytokines or DAMPs/PAMPs receptors induces apoptosis or cell cycle arrest^34^.

Despite advances in medical interventions, including CRRT, SIAKI has been reported to be associated with a high mortality rate of 50–60%^3,32^. Until now, most studies on the effect of CRRT on mortality in patients with SIAKI have focused on the timing and dose of CRRT^32^ and little research has been conducted on the association between survival and fluid overload in patients with SIAKI receiving CRRT. In this study, we investigated the association between fluid overload and survival in patients receiving CRRT considering the important role of fluid balance in patients with SIAKI.

Most previous studies on fluid overload in patients with AKI receiving or not receiving RRT have included a heterogeneous population of patients with AKI, including both SIAKI and non-SIAKI^9–15^, whereas only patients with SIAKI were included in the present study. To the best of our knowledge, the present study is the first to investigate the association between fluid overload and survival in patients with SIAKI receiving CRRT. Previous studies confirming the adverse effects of fluid overload on survival in patients with AKI used various definitions of the degree of fluid overload, including a percentage of fluid accumulation > 10% over the baseline weight^12,13,15^. However, fluid overload > 10% over the baseline weight was arbitrarily defined without any basis for its definition, and the best cutoff value of the degree of fluid overload for predicting mortality was unknown. In this study, we divided fluid overload into fluid overload from AKI diagnosis to CRRT initiation (%FOpreCRRT) and total fluid overload from AKI diagnosis to ICU discharge (%FOtotal, %FOpreCRRT + %FOpostCRRT) and found that %FOpreCRRT > 4.6% (AUC, 0.826; P < 0.001) and %FOtotal > 9.6% (AUC, 0.834; P < 0.001) were the best cutoff values of the degree of fluid overload for predicting the 28-day mortality. We believe that these cutoff values could help guide fluid management in critically ill patients with SIAKI receiving CRRT and conduct further research on the association between fluid overload and survival in these patients.

In the present study, we demonstrated that both %FOpreCRRT and %FOtotal were independent risk factors for the 28-day mortality in patients with SIAKI receiving CRRT, and the results of our study are in line with those of previous studies. However, most previous studies have only assessed fluid accumulation either before RRT^10,14,15^ or during the period of ICU or hospital stay^11,12^. In contrast, the present study assessed both %FOpreCRRT, in which initial fluid resuscitation was implemented in patients with SIAKI, and %FOtotal, in which the effect of CRRT on fluid overload was reflected. Thus, our study clarified the association between fluid overload and survival in these patients. Finally, the multivariable analysis of our study demonstrated that patients with %FOpreCRRT > 4.6% and %FOtotal > 9.6% were 1.9- and 3.37-times more likely to die within 28 days after ICU admission than those with %FOpreCRRT ≤ 4.6% and %FOtotal ≤ 9.6%. Furthermore, this association between survival and fluid overload was consistent across various subgroups, including the high SOFA group (> 14 points) or low SOFA group (≤ 14 points), late CRRT group (> 1.5 days) or early CRRT group (≤ 1.5 days), age > 65 or ≤ 65 years, male or female sex, diabetes or no diabetes, and oliguria or no oliguria, suggesting a robust effect of fluid overload on survival in patients with SIAKI receiving CRRT.

One of the major findings is that minimizing fluid overload by CRRT reduced mortality in critically ill patients with SIAKI. In previous studies, the randomized evaluation of normal versus augmented level (RENAL) of replacement therapy study investigators showed that negative fluid balance during CRRT was associated with decreased mortality in patients with AKI (n = 1453)^7^. Murugan et al. showed that achieving a high intensity of net ultrafiltration (> 25 mL/kg/day) through CRRT or intermittent RRT was associated with lower 1-year risk-adjusted mortality in patients with AKI (n = 1075), confirming the importance of RRT in fluid management in these patients^8^. Recently, Hall et al. demonstrated that a decline in fluid accumulation through CRRT was associated with a lower risk of mortality in critically ill patients with AKI (n = 820)^17^. In the present study, the 28-day mortality was the highest in Group 4 (84.7%, %FOpreCRRT > 4.6% and %FOtotal > 9.6%), followed by Group 3 (65.0%, %FOpreCRRT ≤ 4.6% and %FOtotal > 9.6%), Group 2 (43.6%, %FOpreCRRT > 4.6% and %FOtotal ≤ 9.6%), and Group 1 (22%, %FOpreCRRT ≤ 4.6% and %FOtotal ≤ 9.6%). The multivariable analysis also showed that people in Group 4, Group 3, and Group 2 were nearly six times, four times, and two times more likely to die than those in Group 1. These findings showed that minimizing the fluid overload using CRRT is associated with reduced mortality in critically ill patients with SIAKI. One can argue that tolerating fluid removal using CRRT is a sign of less severe illness, such as hemodynamic stability. Thus, our results need to be interpreted cautiously regarding whether the beneficial effect of fluid removal using CRRT on mortality is owing to its direct therapeutic effect or the fact that patients with less severe illness could tolerate fluid removal. However, our multivariable analysis showed that the association between survival and fluid overload remained significant after adjustment for disease severity indices such as SOFA score, APACHE II score, vasopressor use, ventilator dependency, and oliguria, suggesting that disease severity alone may not account for our findings. Finally, in clinical practice, clinicians are inevitably confronted with fluid overload during the management of patients with SIAKI, and the results of our study suggest that efforts are needed to maintain %FOpreCRRT ≤ 4.6% and %FOtotal ≤ 9.6% as much as possible to improve the survival of patients with SIAKI receiving CRRT.

One of the strengths of our study is the inclusion of time from AKI diagnosis to CRRT initiation in the multivariable analysis. We found that a 1-day increase in the time between AKI diagnosis and CRRT initiation was associated with an 11% increased risk of the 28-day mortality in our study population. Whether early initiation of CRRT improves mortality in critically ill patients with SIAKI remains controversial, and previous studies have reported inconsistent results. Several retrospective studies have shown that early initiation of CRRT has survival benefits in critically ill patients with SIAKI^35–38^. However, two randomized clinical trials reported that early application of CRRT is deleterious^39^ or there was no difference in mortality between early and late RRT initiation in these patients^40^. This discrepancy between the results of previous studies might be owing to an unstandardized definition for early and late initiation of CRRT. Furthermore, the optimal timing to initiate CRRT remains undefined in patients with SIAKI. In the present study, we found that the best cutoff value for the time from AKI diagnosis to CRRT initiation for predicting the 28-day mortality was > 1.5 day, with a sensitivity of 71.3% and specificity of 75.6% (AUC, 0.808; *P* < 0.001). The Kaplan–Meier curve also showed a significant difference in the 28-day mortality between the late CRRT group (> 1.5 days) and early CRRT group (≤ 1.5 days) (Supplementary Fig. S3). Despite the retrospective design of our study, this observation suggests that CRRT initiation within 1.5 days from AKI diagnosis, if possible, should be encouraged to improve the survival of critically ill patients with SIAKI.

Despite its strengths, our study had some limitations. First, owing to its retrospective design, it is not possible to discern whether fluid overload is solely a marker of more severe illness or a causal contributor to mortality in our study subjects. However, as discussed above in the present study, we attempted to adjust for the disease severity indices, such as the SOFA score, APACHE II score, vasopressor use, and ventilator dependency, and found that fluid overload during CRRT was independently associated with the 28-day mortality, suggesting that fluid overload is a potentially modifiable risk factor for mortality in patients with SIAKI receiving CRRT. Second, we included a specific subset of critically ill patients, namely those with SIAKI who received CRRT. Thus, selection bias could not be avoided, and the results of our study might not be generalizable to other populations of critically ill patients with AKI. Third, fluid management using CRRT was implemented through discussion and consultation with the attending nephrologist without a standardized protocol. Thus, variations in fluid management might have affected the effect of fluid overload on survival in the present study.

In conclusion, our study demonstrated that %FOpreCRRT > 4.6% and %FOtotal > 9.6% were the best cutoff values of the degree of fluid overload for predicting the 28-day mortality in critically ill patients with SIAKI receiving CRRT. Both %FOpreCRRT and %FOtotal were independent risk factors for the 28-day mortality. Future randomized controlled studies are needed to confirm whether fluid overload is solely a marker of more severe illness or a causal contributor to mortality and whether minimizing fluid overload using CRRT improves survival in critically ill patients with SIAKI.

## Supplementary Information

Supplementary Information.

## Data Availability

The datasets used and/or analyzed during the current study are available from the corresponding author on reasonable request.
