# Supplementary material for: Effect of fluid overload on survival in patients with sepsis-induced acute kidney injury receiving continuous renal replacement therapy
Source: Sci Rep. 2023 Feb 16;13:2796. doi: 10.1038/s41598-023-29926-w (PMC9935605; doi:10.1038/s41598-023-29926-w)
Supplement: Supplementary file 1 — Supplementary Information. [file 41598_2023_29926_MOESM1_ESM.docx]

**Effect of fluid overload on survival in patients with sepsis-induced acute kidney injury receiving continuous renal replacement therapy**

Il Young Kim^1,2^, Suji Kim^1,2^, Byung Min Ye^1,2^, Min Jeong Kim^1,2^, Seo Rin Kim^1,2^, Dong Won Lee^1,2^, Hyo Jin Kim^1,3^, Harin Rhee^1,3^, Sang Heon Song^1,3^, Eun Young Seong^1,3^, and Soo Bong Lee^1,2*^

^1^Department of Internal Medicine, Pusan National University School of Medicine, Yangsan, South Korea ^2^Research Institute for Convergence of Biomedical Science and Technology, Pusan National University Yangsan Hospital, Yangsan, South Korea

^3^Medical Research Institute, Pusan National University Hospital, Busan, South Korea

^*^Correspondence: E-mail: [sbleemd@pusan.ac.kr](mailto:sbleemd@pusan.ac.kr)

**Fig. S1** Flow diagram for patient enrollment.


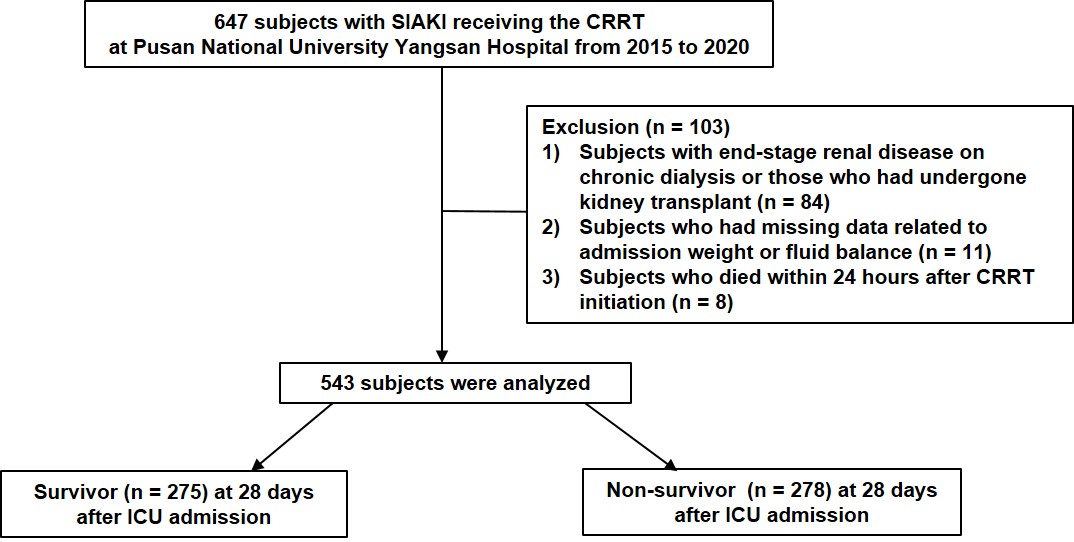


CRRT, continuous renal replacement therapy; ICU, intensive care unit; SIAKI, sepsis-induced acute kidney injury

**Fig. S2** Receiver-operating characteristic curves for SOFA score (a) and time from AKI diagnosis to CRRT initiation (b) for predicting 28-day mortality in the study population (n = 543).


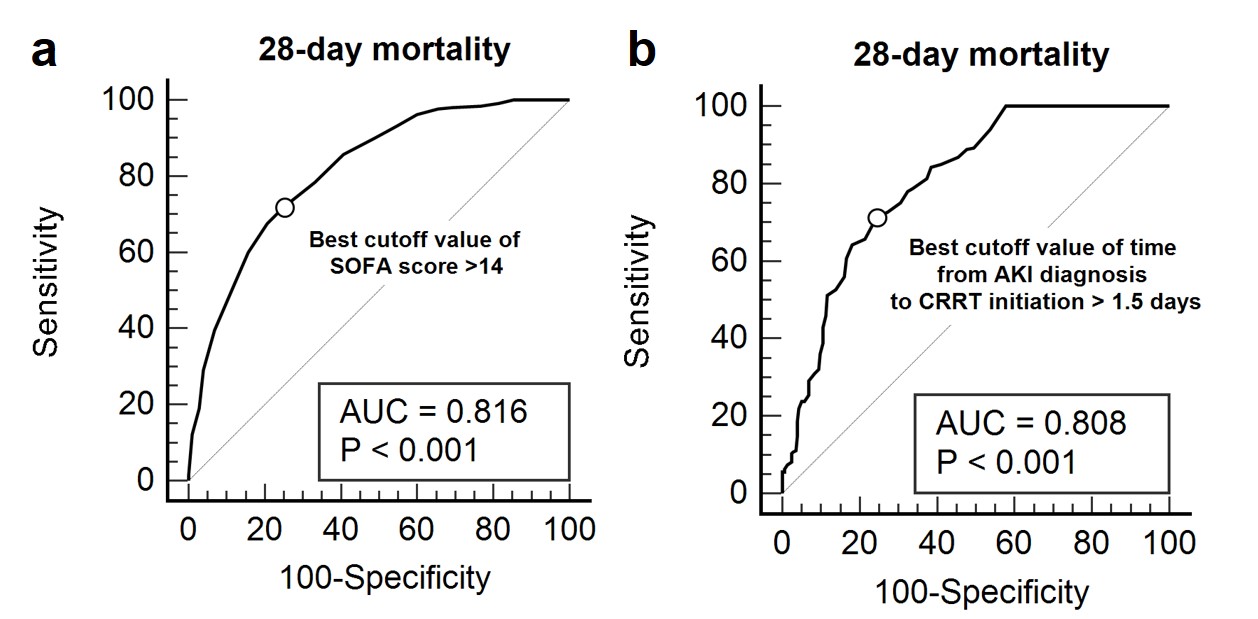


The AUCs were 0.816 (95% CI: 0.781–0.848, P < 0.001) for the SOFA score and 0.808 (95% CI: 0.773–0.841, P < 0.001) for the time between AKI diagnosis and CRRT initiation. The best cutoff value for the SOFA score was >14 points, with an associated sensitivity of 72.0% and specificity of 74.9%. The best cutoff value for time from AKI diagnosis to CRRT initiation was >1.5 days, with an associated sensitivity of 71.3% and specificity of 75.6%.

AKI, acute kidney injury; AUC, area under the curve; CI, confidence interval; CRRT, continuous renal replacement therapy; ICU, intensive care unit; SOFA, Sequential Organ Failure Assessment

**Fig. S3** Kaplan–Meier survival estimate according to the best cutoff value of time from AKI diagnosis to CRRT initiation in the study population.


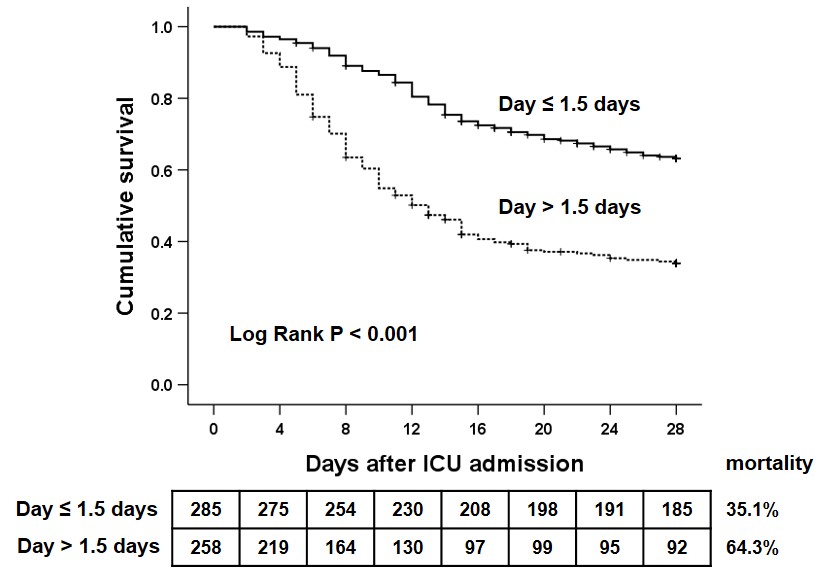


There was a significant difference in survival among patients who received CRRT early (≤ 1.5 days) or late (> 1.5 days). AKI, acute kidney injury; CRRT, continuous renal replacement therapy; ICU, intensive care unit.
